# Climate Change, Health Risks, and Vulnerabilities in Burkina Faso: A Qualitative Study on the Perceptions of National Policymakers

**DOI:** 10.3390/ijerph18094972

**Published:** 2021-05-07

**Authors:** Raissa Sorgho, Maximilian Jungmann, Aurélia Souares, Ina Danquah, Rainer Sauerborn

**Affiliations:** 1 Heidelberg Institute of Global Health (HIGH), Heidelberg University Hospital, Heidelberg University, Im Neuenheimer Feld 324, 69120 Heidelberg, Germany; aurelia.souares@uni-heidelberg.de (A.S.); ina.danquah@uni-heidelberg.de (I.D.); 2 Heidelberg Center for the Environment (HCE), Heidelberg University, Im Neuenheimer Feld 404, 69120 Heidelberg, Germany; jungmann@momentumnovum.com

**Keywords:** climate change, adaptation, health, policymakers, food security, agriculture, West Africa

## Abstract

Climate change (CC) constitutes one of the greatest threats to human health, and requires political awareness for effective and efficient adaptation planning. This study identified the perceptions of climate change and health adaptation (CC&H) among relevant stakeholders, decision-makers, and policymakers (SDPs) in Burkina Faso (BF) by determining their perceptions of CC, of related health risks and vulnerabilities, and of CC impacts on agriculture and food security. We carried out 35 semi-structured, qualitative in-depth interviews with SDPs, representing national governmental institutions, international organizations, and civil society organizations. The interviews were analyzed using content analysis. SDPs shared similar perceptions of CC and concurred with three ideas (1) CC is a real and lived experience in BF; (2) the population is aware of climatic changes in their environment; (3) CC is intertwined with the agricultural and economic development of the country. SDPs identified biodiversity loss, floods, droughts, and extreme heat as posing the highest risk to health. They elaborated five exposure pathways that are and will be affected by CC: water quality and quantity, heat stress, food supply and safety, vector borne diseases, and air quality. In conclusion, SDPs in Burkina Faso are highly aware of CC hazards, relevant health exposure pathways, and their corresponding health outcomes. Mental health and the interplay between social factors and complex health risks constitute perception gaps. SDPs perceived CC&H risks and vulnerabilities align with current evidence.

## 1. Introduction

Climate change (CC) is widely acknowledged to occur through the accumulation of greenhouse gases in the atmosphere, arising from the combustion of fossil fuels and changing land use. Although the climate has always been changing, human-induced changes are a significant contributor to the drastic and accelerating changes we are witnessing [1,2,3]. Consequently, today, CC constitutes one of the greatest threats to humankind, as it will impact everything from population growth to environmental degradation, and regional security to nation development across the globe [4,5,6,7,8].

Widely recognized, the increasing impact of CC on developing nations is a focal action point internationally for academic and policy communities [9,10]. In this study, we applied the Intergovernmental Panel on Climate Change (IPCC) definition of CC “*A change in the state of the climate that can be identified (e.g., by using statistical tests) by changes in the mean and/or the variability of its properties and that persists for an extended period, typically decades or longer*.” The lived experiences of CC were discussed through climate variability, defined as “*variations in the mean state and other statistics (ex. the occurrence of extremes) of the climate at all spatial and temporal scales…”*. [11].

Low- and middle-income countries (LMIC), although overall emitting only low levels of greenhouse gasses, will be disproportionately affected by CC with consequences across most sectors, notably human health and agriculture. Climate change and health (CC&H) issues are especially relevant for West African countries, which are also predicted to experience CC impacts on both agriculture and food security [12,13]. CC and climate variability processes lead to changes which countries must adapt to, making the preparation, framing and implementation of CC policies increasingly essential for LMICs [14,15].

Current climate models research project significant changes in rainfall patterns and gradual temperature increases in West Africa [16,17]. Taking 1971–2000 as reference years, the five regional climate models of Ibrahim et al., 2014 forecast delayed rainy seasons, lengthened dry spells, decreasing rainfall events (0.1–5 mm/d), and more extreme rainfall events ([50 mm/d) [18]. In Burkina Faso, the temperature is projected to be 2 °C degrees higher by 2100 compared to 1990–2000 average [19,20]. In Tanzania, a modeled seasonal temperature increase of 2 °C projected reduced average yields for important staple crops, such as maize (−13%), sorghum (−8.8%) and rice (−7.6%) by 2050 [21]. In addition to the higher temperature, reduced water availability and increased evaporation are to be expected.

In addition to affecting agricultural yields and water resources, there are health risks associated with CC. CC affects human health in a multitude of ways, through exposure pathways [22,23]. CC affects health directly and indirectly, from higher morbidity and mortality of elderly populations linked to heat, heat stress and heat waves [24,25,26,27] to under-nutrition in young children due to changes in rainfall and yields [28,29]. In 2016, Bunker et al. carried out a systematic review and meta-analysis of the impact of heat on the elderly in low and middle-income countries. The authors concluded that a 1 °C temperature rise increased cardiovascular, respiratory, and cerebrovascular mortality by 3.44%, 3.60% and 1.40% respectively [25]. Another modeling study by Nelson et al. rojected that by 2050, there will be an additional 25.2 million children under five years of age, whose stunting can be attributed to climate change. Approximately 10.5 million of these children will be in sub-Saharan Africa [30].

Burkina Faso is particularly susceptible to CC impacts on health: Its population is highly exposed (reliant on small-scale subsistence farming), severely vulnerable (low adaptive capacity), and faces CC hazards (mini-droughts, heatwaves, torrential rainfalls, and extreme weather) [19,31,32]. In addition, both the individual farming households as well as the nation at large have limited adaptive capacity.

Changes to rainfall and yields contribute to one of Burkina Faso’s greatest CC&H vulnerabilities, which lies at the intersection of CC, nutritional and food security, due to the relationship between precipitation and agriculture [12,13]. As CC alters precipitation patterns, the country’s agricultural sector, which employs and serves as a means of subsistence for over 70% of the population, is and will continue to be severely impacted [15,33].

To face these effects, stakeholders, decisionmakers and policymakers (SDPs) must strengthen adaptation by planning, implementing, and monitoring CC&H policies [34]. Therefore, the CC perception and political will of those making decisions are key factors in the development and implementation of CC policies [35,36]. Stakeholders (domain experts, development professionals and researchers) shape local, national, and political awareness, while policymakers and decision-makers (actors, politicians and implementation authorities) write and enforce policies [37,38]. Although there is limited literature on the subject, SDP perception, awareness, and knowledge of CC or and the lack thereof has been shown in studies at the local and regional government level in South Africa to influence the prioritization of CC adaptation on the political agenda [39,40]. In Burkina Faso, although SDP are key in the policy process, little is known about their perceptions and understanding of how CC affects health. Therefore, a first step to determine where Burkina Faso lies in the policy process in regards to CC&H is gauging the perception and understanding of the nation’s stakeholder, decisionmakers and policymakers on the issue.

This study aimed to evaluate SDPs’ understanding of CC&H by (i) determining their perceptions of climate change, (ii) defining SDPs’ perceptions of CC&H risks and vulnerabilities, and (iii) identifying SDPs’ perception of CC impact on agriculture and food security in Burkina Faso.

## 2. Materials and Methods

We used an inductive approach, moving primarily from data to theory formation. This best suited the work as there is limited literature on perception of SDPs in West Africa on the subject of CC human health, agriculture, and food security. The frame for analyzing the interviews was built from the descriptive keywords and codes generated from the initial reading of the data. No pre-conceived categories were utilized [41]. Inductive category development was conducted through conventional content analysis for the data analysis [42].

### 2.1. Data Collection

SDPs were defined as individuals with national-level expertise and experience in at least one of four fields: environment, agriculture, animal husbandry, or health. We included study participants, working (i) in national government institutions (environment, agriculture, forestry, or health ministries), (ii) international organizations (United Nations agencies and non-governmental organizations), or (iii) civil society organizations (nationally represented community-based groups and research institutions). These were identified through relevant policy documents, a key informant, or by snowballing with selected participants. We focused on professionals with the capacity to influence, direct, and draft policies, and/or the authority to sign and pass legislation.

In Ouagadougou, the principal investigator (RSo) conducted semi-structured in-depth interviews, in French, in April 2019. The interview guide, developed over three months, consisted of open-ended questions followed by probes and prompts tested and adjusted through three pilot interviews. The interviews were conducted in person and audio recorded. Participants were invited via a letter, one week prior to their interview they received a study information form. Written consent was administered on the day of the interview.

Most interviews were conducted, following the traditional in-depth interviews method, with only the interviewer and one participant. On three occasions, participants requested to ask a colleague to join for the interview. In all three cases, the colleague worked at the same institution in the same or connected area. The new participant gave their informed consent to participate and was enrolled in the study on the spot as a participant. The two respondents then took turns in answering the questions. The interviews lasted 30 to 75 min. At the end of each day, voice memos about the interaction with the participants and interview highlights were recorded. The investigator listened to the recorded interviews weekly and wrote memos reflecting on progress and evaluating needs for further data collection. This process determined information saturation, the basis for deciding when the size of a qualitative sample is considered sufficiently large [43].

All procedures of the present study adhered to the latest version of the Helsinki Declaration. The ethics committee of Heidelberg University [Identification Number: S-594/2018] and the Ethical Committee of the Centre de Recherche en Santé de Nouna (CRSN) [Deliberation Number N2018-013/CIE-CRSN] reviewed and approved the study protocol and study tools. Furthermore, all participants gave oral and written consent for the anonymous publication of study results.

### 2.2. Data Analysis

The recorded interviews were conducted in French and transcribed in French by RSo. In the data analysis process only, the quotes displayed in the manuscript were translated into English. The resulting transcripts—stripped of identifying information, replaced with study specific identification codes—were analyzed in the NVivo 12 Analysis software (QSR International). The transcripts went through a two-cycle coding process resulting in the coding tree. Two researchers (RSo and MJ) conducted the first-cycle descriptive topic coding inductively creating preliminary codes (tree leaves). (RSo) then conducted second-cycle coding for category development [42,44]. All 35 interviews underwent the same process. We developed categories and analyzed their content thematically to assess the linkage between codes and overarching themes (tree branches), aiming to identify cross-cutting themes in the SDPs’ perceptions of CC&H, with additional focus on CC, agriculture, and food security (tree trunk).

### 2.3. Data Validation

Two processes ensured the validity, reliability, and transferability of the research and the production of a high-quality paper [45]:

(1) To enhance the validity of the data, the investigator conducted a dissemination trip to Burkina Faso before publishing study results [46], presenting preliminary results to half of the study participants individually or in pairs and allowing them to provide feedback on the accurate representation of findings. The participants validated the study results prior to publication.
(2) The implementation of the 32-item checklist of the Consolidated Criteria for Reporting Qualitative Research (COREQ) (Supplementary Material 1) ensured that key information about the study (research team, study design, participants, data analysis, reporting) was provided to the reader [47,48]. This information is essential for readers and scientists to assess the transferability of our study to other contexts. Data source comparison was conducted using interviews, daily observations, and weekly memos, in combination with researcher investigator triangulation (between RSo and MJ) through the first cycle coding of the transcripts, to bring together differing perspectives [49,50] and further ensure transferability [51].

In the results section of this paper, participants’ perceptions are illustrated with direct quotes selected for their representativeness and poignancy [52,53]. Each quote is identified with the participant study number (P), branch of work: national government (NG), international organization (IO), or civil society (CS), and age (years).

## 3. Results

**Figure 1:**
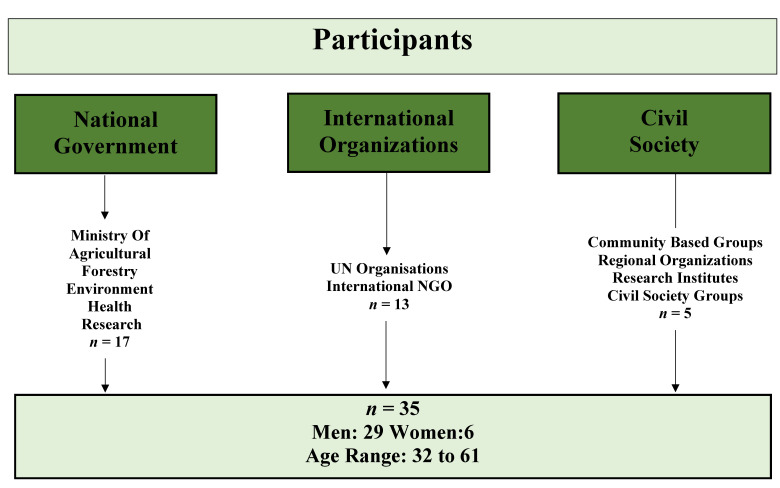
Composition of the participant sample.

The 35 SDPs were aged between 32 and 61 years; 29 participants were male and six were female (Figure 1). The section below provides the results of the in-depth interviews’ structured impact on agricultural and food security.

### 3.1. Perceptions of Climate Change

Regardless of their area of expertise, position, or institution, the SDPs shared similar perceptions and concurred with three ideas. SDPs expressed that, first, CC is no longer theoretical; it is a phenomenon currently experienced in Burkina Faso. Second, the country’s population at all levels is aware of climatic changes in their environment. Third, CC is now undeniably intertwined with the agricultural and economic development of the country.

Answering the opening question “What do you think of climate change?” participant (P03_NG_32) stated: “*We are actually experiencing climate change today, it is no longer at the level of thinking, the facts are there*” and agreed with a United Nations REDD-Plus (The REDD-Plus is a UNFCCC created framework from the Warsaw Conference of Parties to guide activities in emission reduction and sustainable management especial in the forestry sector-https://unfccc.int/topics/land-use/workstreams/redd/what-is-redd, accessed on 8 September 2020): Rural participation diagnostic survey, which found that “*even in the most remote corners, there is an awareness of climate change. For example, if we just ask some people in the villages what the environment was like twenty years ago and how the environment is today, they will say* “*ha!, things have changed, seriously things have changed*”…” (P03_NG_32). SDPs agreed that even in communities where individuals had no specific knowledge of CC and its terminology, they could describe the effects of the phenomenon on their everyday life, their livelihood, and their environment. Most SDPs emphasized that because of its multifaceted impact, CC is connected and intertwined with population and country development. “*When we say CC, we end up saying it’s everything, it’s… threatening the sustainability of our development, because the economic activities of our countries are strongly based on production, exploitation, and management of resources*” (P26_IO_60) stated, adding that resource management and agriculture are primary activities for the economy and for subsistence. Another SDP tied climate fight to political action *“… the fight against climate change, desertification, poverty is the same fight… we can’t dissociate that. You have to have a political approach to be able to address the problems linked to agriculture”* (P30_IO_56).

### 3.2. Perceptions of Climate Change: Health Risks and Vulnerabilities 

**Figure 2:**
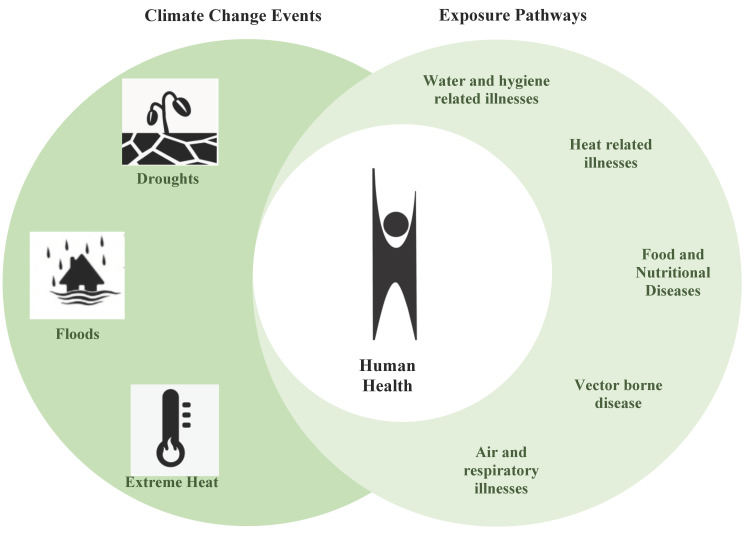
Illustrates how SDPs perceive the overlap between CC events and health exposure pathways at the intersection of human health. The structure of the figure is adapted from the US Environmental Protection Agency 2016 Climate Change and Health Assessment [54].

All SDPs viewed CC&H as undeniably interconnected. They identified extreme heat, droughts, floods, and biodiversity loss as the climatic hazards faced by populations in Burkina Faso leading to five health exposure pathways affected by CC: Water quality and quantity, heat stress, food supply and safety, vector borne disease and ecology, air quality. Lastly participants discussed the importance of medicinal plants in connection to biodiversity loss and the five health exposure pathways along with a multitude of health outcomes (Figure 2).

(1) Water quality and quantity—In terms of CC impact on human health, SDPs mostly mentioned the effect on water and hygiene-related illnesses due to (i) decreasing water quantity/water sources (man-made and natural), (ii) decreasing personal hygiene and water consumption, and (iii) increasing illnesses such as cholera, malaria, and diarrheal diseases.

Some SDPs pointed to high levels of water evaporation as well as lower precipitation as key reasons for both natural watering points (holes, ponds) and man-made reservoirs across the country drying up early in the dry season. *“Who says drought, also says decreasing precipitation so there is less water and when there is less water, we observe less hygiene and …. we get sick, especially diarrheal diseases, which affect children”* (P14_IO_62) Reduced water availability creates new problems: *“… in the rivers, if there are bacteria then grows the concentration of these bacteria. And if it’s the main source of water, people drink that water. Due to bacterial concentration, they get sick more often and [are] faced with health consequences. And if it’s cholera, it’s dramatic because cholera kills…”* (P14_IO_62) Water contamination was also referenced in relation to chemical leaks that make water non-potable. These difficulties emerge as a result of flooding as well as lack of water. *“…The malaria rate has increased a little, since there have been a lot of flooded areas… As you see, the sanitation system is not so great… gutters are blocked and become mosquito nests, leading to a large proliferation…”* (P30_IO_56).

(2) Heat Stress—SDPs outlined the multiple impacts of extreme heat, which (i) affects body thermo-regulation, (ii) creates physical discomfort, (iii) reduces work capacity, and (iv) increases elderly and infant vulnerability.

(P15_IO_56), whose work focuses on desertification, explained that the current levels of heat in Burkina Faso today match those recorded in the middle of the Sahara desert 15 years ago, indicating that this level of heat is unprecedented. Such intense heat affects the overall thermic regulation of the human body. *“If I take the Sahel region, where children during a period of the year have one additional degree on their body temperature which is 37°C, when a child is at 38°C in the Sahel region in the middle of the day, in general the health personnel does not worry. This means that [heat] has an impact on thermal regulation, which is already a problem.”* (P15_IO_56) He continued to explain that in the Sahel region body temperature increases as high as one degree are now to be expected along with decreased capacity for labor, as heat directly affects the physical work capacity of a population and causes dehydration, irritation, and burning of skin. SDPs also emphasized the effects on vulnerable groups: *“the elderly, infants are vulnerable [to these extreme temperatures] in the sense that they become dehydrated very quickly. We observe deaths, and cardiovascular diseases and respiratory disorders and these elderly people are very sensitive to that… in our country when there are these terrible heatwaves the elderly are the most affected, they are more likely to die. Unfortunately, at the level of our African countries we are not yet keeping exact statistics …”* (P14_IO_62) Lastly, he highlights that Burkina Faso, like many other African countries, does not keep precise statistics on the effect, consequences, and outcome of extreme heat and heatwaves on the population.

(3) Food Supply and Safety—When discussing food and nutrition, SDPs listed the following CC-related obstacles: (i) decreasing agricultural output, (ii) decreasing food availability, and (iii)) decreasing food variety and nutritional value.

In Burkina Faso, “*Food is considered the first medicine*” (P09_NG_55), and being well nourished is considered primary prevention against the onset of disease or illness. SDPs easily linked climate change effects and nutrition *“… drought necessarily means reduction in food production. When one says reduced food production, one says insufficient food, insufficient food, says malnutrition, therefore weakening of the state of health and when one says weakening of the state of health one says susceptibility to diseases…”* (P14_IO_62) SDPs also linked food security to support food and nutrition counseling efforts *“… food security… is something that has an impact on nutrition. We have given nutritional advice to families or the population, and knowledge about the nutritional values of different foods. If this [food] is not available it causes difficulties”* (P15_IO_56).

(4) Vector-borne disease and ecology—(i) Mosquito resistance and (ii) flooding and sanitation conditions were resounding concerns especially regarding malaria, cited by multiple SDPs as the greatest health concern in Burkina Faso, negatively affected by CC-related events.

Participants also expressed concern about increasing mosquito resistance to chemical insecticides used in farming: *“…Just in the slightest zone of humidity, these larvae develop and this actually worsens diseases among these populations.”* (P12_NG_60) They also mentioned that flooding and poor sanitation are creating favorable conditions for mosquitoes. *“The more the floods create breeding places conducive to the multiplication of mosquitoes, which cause certain illnesses in particular malaria, dengue … [they also create] the multiplication of vectors and illnesses including schistosomiasis, since the mollusks need water”* (P15_IO_56). Other illnesses were mentioned. “…*if there is a flood, there is a lot of water, there is a risk of cholera! Also, there are certain hemorrhagic diseases like zika, dengue, Ebola which have a link…”* (P15_IO_56).

(5) Air Quality—SDPs pointed to heat and evaporation as a cause of increased dryness and dust in the country: “…drought makes … a lot of dust, exposing [people] to certain respiratory infections, infections in the nose, throat and also conjunctivitis.” (P15_IO_56) Aches and discomforts can lead to irritation, infection, and inflammation throughout the respiratory tract and other parts of the body “…meningitis… occurs in what is called the meningitis belt…where the rainfall is the lowest compared to the rest of the territory outside this zone…” (P15_IO_56). SDPs foresee respiratory illnesses increasing, especially in the city, where “atmospheric pollution in Ouagadougou can be seen” (P13_NG_61) and vehicle numbers are rapidly increasing. “Go to a family these days, you can see at least three or four vehicles; each child has a motorcycle, the parents maybe a car, all of these moving engines increase the rate of [pollution] production. And we know that the release of CO_2_ leads to the deterioration of the atmosphere and that will have an impact on health.” (P13_NG_61).

In connection to the five health impacts, SDP extensively discussed medicinal plants, centering the conversation on (i) the utility of traditional medicine, (ii) the importance and high value of medicinal plants for local communities, and (iii) the observed biodiversity loss due to CC.

Participants emphasized the importance of traditional medicine stating “There is a WHO study which found that 80% of the populations in developing countries are treated with biodiversity, [local] plant-based treatments. So, the fact that the plants are disappearing is a problem. Because people in Burkina Faso and in developing countries cannot afford laboratory drugs.” (P09_NG_55). As a result of the loss of biodiversity “… there are [traditional medicine] plants that [populations] can no longer find or they have to go further to find them to be able to treat themselves.” (P17_NG_54) The Burkina Faso government has recognized the importance of traditional medicine and “are in the process of integrating this traditional medicine, alongside modern medicine… The ministry has a law that recognizes traditional medicine and which integrates traditional medicine.” (P17_NG_54). According to participants, to keep this initiative going, one must keep traditional medicine plants alive and accessible. These plants are disappearing in Burkina Faso due to the CC which is affecting plant biodiversity and survival.

One SDP shared the story of a successful herbalist who provides affordable community health care where few can afford hospital treatment. *“You have Doctor [NAME’s] pharmacy, which only uses local plants… So, he has contracts with people who deliver local plants, which he transforms to treat the population.”* (P05_NG_59) Essential to cover the population’s food and medical needs, especially for people who cannot afford western medicine, the local plant forest must be kept alive and accessible.

### 3.3. Perceptions of Climate Change: Agricultural and Food Security Impacts

#### 3.3.1. Agriculture

All 35 SDPs linked CC with agriculture because, in their perspectives, CC is felt first and foremost through changes in rainfall on agricultural output. *“…we feel it starkly in Burkina Faso. Firstly, in terms of rainfall, because it has been noted that in the past, the rain was harnessing well in time and space, but given this climate change, there are periods of pockets of drought, sometimes there are floods… we can see that the seeds that we used are no longer adapted to the new situation, hence the need to adapt all of this.”* (P32_IO_32).

On agricultural production in Burkina Faso, SDPs expressed one of two opinions: (i) nationally, agricultural production was seen in a see-saw pattern or (ii) agricultural production has been declining. They unanimously agreed that (a) adaptation to CC is essential for continued agricultural productivity in the country and (b) the pace of adaptation is too slow throughout the country.

Discussing agricultural production in the past two years, between 2017 and 2018, (P32_IO_32) stated “The agricultural situation is going up and down. There are years where things are going well, there are years where things are not going well. For example, last year, on the whole, we produced well even if we did not cover 100% of cereal needs …” (P32_IO_32). Pluviometry is a primary factor for production. Many SDPs mentioned unequal rain distribution across regions. “We have regions that are in deficit and we have surplus regions that very often do not manage to make up the cereal deficit, so that means that our agriculture is subject to climatic hazards.” (P31_NG_40) “In terms of adaptation, I think there is a lot of change, but actually, it is slow…, But people see for themselves that if they do not change their way [of cultivating], they will lose. So gradually, there are changes taking place…, for example digging Zaï for agricultural production.” (P32_IO_32).

#### 3.3.2. Food Security

Participants’ perceptions of Burkina Faso’s food security (availability, access, utilization, and stability) varied greatly [55,56,57]. There was no consensus on the country’s current food security status.

The majority expressed that it was unstable to some degree “… food security in Burkina Faso remains fragile, due to a certain number of constraints, which are more or less natural, physical, linked in particular to the environment and climate change.” (P19_IO_50). Furthermore, interviewees stated that the fragility of food security at national level was reflected in insecurity at household level. A few SDPs perceived increased stability in food security, while an equally small fraction stated the situation was either slowly improving or heavily deteriorating.

One participant linked the nation’s food insecurity to undernutrition: “[The nutritional situation in Burkina Faso] has gone from fairly high rates of acute malnutrition, chronic malnutrition, and underweight which were also quite high, to today–the latest data of 2018 showing [the figures] halved compared to 12 years ago. But despite that, we are not yet at acceptable rates according to institutions such as the WHO and UNICEF” (P15_IO_56).

## 4. Discussion

We explored the SDPs’ perceptions of climate change, as well as CC&H risks and vulnerabilities, and identified more specifically their perceptions of agriculture and food security. The SDPs’ perceptions converged on three ideas: CC is a lived reality in Burkina Faso, all tiers of the population are aware of climatic changes in their environment, and CC is now undeniably intertwined with the development of the country. SDPs identified biodiversity loss, floods, droughts, and extreme heat as the climatic events posing the highest risk to human health in Burkina Faso. Although they agree on the lived experience of CC, they did not unanimously agree on the current status of food security in Burkina Faso, especially in comparison with the past decade. They expressed that agricultural adaptation is essential and that the pace of adaptation is too slow throughout the country. SDPs listed five health exposure pathways affected by climate change: Water quality & quantity, heat stress, food supply & safety, vector borne distribution and ecology, and air quality.

The study findings should be interpreted with caution based on the following study strength and limitation. Arguably the gender imbalance of our interviewee sample could be considered a limitation. However, women in our study still overrepresent the proportion of women serving in high level (decision-making) positions in Burkina Faso. Women make up the majority of Burkina Faso’s total population but are underrepresented in politics, policy, and decision making [58]. Between 1946 and 2000, 750 men and only 23 women (3%) served as members of the legislature [59]. With the creation of the Ministry for Promotion of Women and Gender and the adoption of legislation such as the 2009 Act No. 010-2009/AN, which sets quotas for legislative and local elections, there has been progress in increasing women’s integration in political parties, in government and in decision making roles, but this progress is slow [60,61].

The active reduction of biases to enhance validity during data collection, data analysis, and results presentation is a strength of the study. The researcher reflected on the data collection experience and personal perspective to limit the introduction of bias [50]. Furthermore, the lead author of the publication, a female doctoral researcher, who lived in the country for over a decade and is accustomed to the country’s culture, was also the sole interviewer [62]. There was no personal connection between the interviewer and the study participants prior to starting the study. Study participants received a study invitation letter, prior to the face-to-face meeting, on the researcher and research institution, to which all participants responded. Participants’ comfort was at the forefront of the interview process. We allowed them to select the time, date, and place for the interviews. Interviews and data analysis were conducted in French, the interviewing language, eliminating the threat to validity associated with poor translation of entire data sets [63]. Only the quotes in this publication were translated into English. The study also ensured, with the COREQ, the inclusion of rich data description in the manuscript, as information on research steps and context are key to the transferability of the research to similar contexts and settings [47,48,51].

### 4.1. Perceptions of Climate Change

Across the published literature, numerous studies have been conducted in high-income countries on the perceptions and understanding of CC by SDPs [64], and a few studies more specifically on CC&H [65]. Some studies have even explored, if and how SDPs interpret and understand CC&H related literature and figures [66]. From LMICs, evidence on SDPs perceptions is limited as the focus of research has been on rural farming/herding [15,67] and urban slum households and populations [68].

In sub-Saharan Africa, some reports based on CC meetings, forums, and workshops involving SDPs on CC [69] are accessible but little exists with regard to scientific or peer-reviewed literature compared to high income countries. The current published papers on SDPs’ perception are from South Africa [39,40] and Ghana [70]. Tetteh et al. [70] studied the understanding of CC impact on smallholder farmers among Ghanaian policymakers. Even though the participants were SDPs, the focus and analysis were still on the farming population. The studies in South Africa [39,40] which investigated the intersection of CC, the environment, and health focused on stakeholders, policymakers, and decisionmakers at the local county level. In fact, in those countries in which SDPs were knowledgeable and aware of CC, an increased number of CC action and programing was seen due to the leadership of the knowledgeable SDPs.

In our study, for the first time, SDPs at the national level of Burkina Faso were interviewed, and our data echo some of the findings from South Africa [37,38]. We found that regardless of their position and institution, SDPs shared similar perceptions on CC. They stated that CC is a phenomenon that is lived and experienced in Burkina Faso, further explaining that this has created a level of awareness across all segments of the population. They also expressed that CC is undeniably intertwined with the agricultural and economic growth and development of the country. The SDPs also concretely identified extreme heat, droughts, floods, and biodiversity loss as the climatic hazards faced by populations in Burkina Faso. These lead to five health exposure pathways conferred by CC: Water quality and quantity, heat stress, food supply and safety, vector borne disease and ecology and air quality, with the importance of medicinal plants underlined as an overarching subject. These health exposure pathways then lead to a variety of outcomes outlined by the SDPs. The SDPs were well aware and highly knowledgeable of both, CC and CC&H. Their ability to perceive and identify the risks and vulnerabilities of CC is the first of many steps in the process of moving the country on the path of CC&H policy and policy action [71,72].

### 4.2. Perceptions of Climate Change, Health Risks and Vulnerabilities: Congruence with Scientific Evidence?

In 2008, the WHO identified five priority areas for research and pilot studies on CC&H: “health vulnerability, health protection, health impacts of mitigation and adaptation policies, decision-support and other tools, and costs of health protection from climate change” [73]. Four out of these five priority areas are key aspects of adaptation, especially for LMICs. WHO member states passed an assembly resolution drawing attention to the necessity of such work, but significant gaps remain, especially regarding quantitative research in developing countries [65]. In their paper “The Imperative for Climate Action to Protect Health”, Haines and Ebi addressed two WHO priority areas: health vulnerabilities and health protection [22] and discussed, how CC&H events, combined with demographic, socioeconomic, and environmental factors, affect human health through seven exposure pathways.

**Figure 3:**
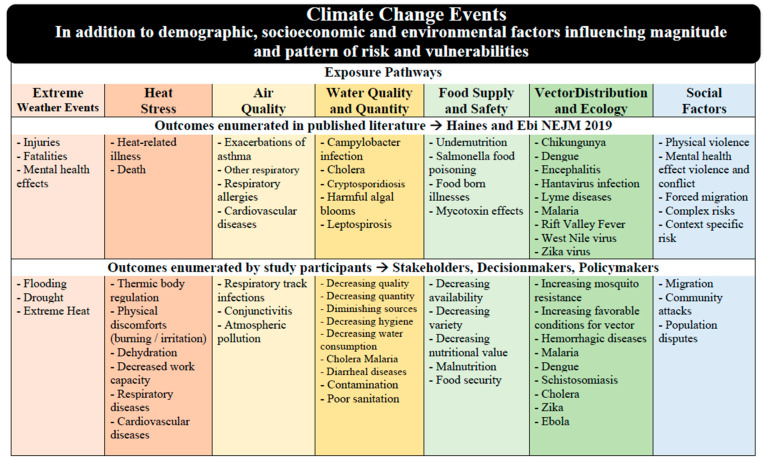
Illustrates and compares results of SDPs’ perceptions (Section 3.1) and published literature (Section 4.2) reviewed by [22].

The CC&H exposure pathways and specific outcomes enumerated by SDPs overlapped largely with those listed by Haines and Ebi (Figure 3). To some degree, the study participants discussed all of the seven exposure pathways—extreme weather events, heat stress, air quality, water quality and quantity, food supply and safety, vector disease and ecology, and social factors. They focused at length on heat stress, water quality & quantity, food supply & safety, and vector-borne disease and ecology, which encompass malaria, meningitis, and cholera. In fact, these conditions are among the main climate-sensitive diseases in Burkina Faso and throughout sub-Saharan Africa [74]. Water and air quality were discussed in relation to both, rural and urban areas, which accords with recent evidence on CC effects in slums in sub-Saharan Africa and Southeast Asia.

At the forefront of SDPs’ CC&H concerns, the interviewees saw the issue of the decreasing availability of medicinal plants in Burkina Faso. These plants are widely used in Burkina Faso for 22 classes of health disorders [75] but were not discussed in Haines & Ebi [22]. Medicinal plants, biodiversity, conservation, and species extinction in times of CC were focal points in the WHO 2015 report “*Connecting Global Priorities: Biodiversity and Human Health*.” [76]. SDPs also expressed concern over the lack of monitoring of the impacts of CC on the health of the population. SDPs highlighted that data and statistics are still not recorded for outcomes such as death resulting from heatwaves, making it difficult for Burkina Faso to properly study the effects of CC on health. This was a particular concern in regards to vulnerable groups such as children and the elderly.

In comparison to the Haines & Ebi 2019, pathways, participating SDPs least discussed extreme weather events and social factors. SDPs discussed CC weather events and highlighted the ones with the highest possible hazard for the population but did not name this as a health exposure pathway. They mentioned extreme weather events as underlying factors, multipliers, and causes of outcomes in individual health and when examining health effects at a population level. This might be rooted in the difficulties to assign one particular weather event to CC [77]. “Social Factors”, the seventh exposure pathway in [22] was not identified as a health exposure pathway by SDPs, although participants enumerated many of the health outcomes which fall under this pathway (Figure 3). In comparison to the outcomes under the “Social Factors” pathways that Haines & Ebi identified, SDPs in this study did not mention mental health or complex risks (Figure 3). These two constituted knowledge. Although complex risks in relation to climate change have been discussed in the sub-Saharan African context [78,79], mental health and climate change still remain understudied. Literature highlighting the pathways between climate change and mental health is growing [80,81,82], but few pertain to the West Africa region [15,83].

### 4.3. Perceptions of Climate Change: Agriculture and Food Security Impacts 

Although SDPs had varied perceptions of the nation’s current food security compared with past decades, they agreed that agricultural production—one component of national food security—was highly vulnerable to climate variability. They viewed adaptation to CC as essential for continued agricultural productivity, particularly for West African countries whose economies and livelihoods rely on arable agriculture and pastoralism [84]. Agriculture employs over 60% of the labor force and provides food supplies to over 70% of the West African population [85]. The fact that a large share of households are self-sufficient based on their rain-fed agriculture and smallholder farming explains why climate variations such as rainfall and drought have severe impacts on West African populations’ livelihoods [86]. SDPs enumerated that, although essential, adaptation is embraced and implemented at a slow pace. Despite the fact that agricultural adaptation methods are known in Burkina Faso, households report an array of barriers—financial and time constraints, material and labor shortages, and inaccessible information—to implementation. SDPs shared farmers’ concerns about agricultural production and food security and, like them, perceived agriculture, food, and health adaptation as key to continued subsistence in Burkina Faso [15]. To help the population overcome adaptation barriers, adaptation planning, policy, and implementation must be incorporated in the country’s sustainable and long-term development planning [15,34,87]. The necessity of combining CC adaptation and development work, echoed by SDPs in this study, is especially important in West Africa where governmental and non-governmental institutions are weakened by restrictive budgets, despite possibilities from streams such as The Global Environment Facility and The Green Climate Fund [88,89].

### 4.4. Recommendation for Research and Policy

Based on our findings, a three-pronged agenda has emerged for research and policy:

(i) Identifying and surveilling vulnerable groups to CC hazards and health outcomes through a two-tier system. Our study highlighted the vulnerability of particular groups to CC hazards and resulting health outcomes, notably the elderly and young children. SDPs discussed the lack of information, data, and statistics collected around the outcomes of CC on the health of the population. We recommend the implementation of a climate-health impact surveillance system with a two-part focus (a) identifying and assisting vulnerable populations in times of CC events such as heat waves and (b) collecting information, data, and statistics on the health outcomes at the populations level, so the relevant institutions can better understand, learn, adapt and react to safeguard human health during future CC events.
(ii) Training of SDPs on mental health consequences and social factors influencing and resulting from CC. This study has identified gaps in SDPs’ perceptions of social factors interaction with CC&H, more specifically regarding mental health and complex risks. To address this minor gap, training relevant stakeholders should be offered. While closing this knowledge gap, the training would also draw attention to these issues, strengthen the expertise of key stakeholders, decision-makers and policymakers and increase their awareness further edging them towards action.
(iii) Investigating where Burkina Faso is in the process of creating a CC&H adaptation policy. This study illustrates that SDPs are knowledgeable about CC&H risks and vulnerabilities, therefore, research should look beyond awareness and towards political will. We suggest investigating if and how this SDP awareness is driving policy to address the issue of CC&H by examining where Burkina Faso is in the process of drafting adaptation policies. Future research through a policy frame should investigate the factors, besides knowledge and awareness which will influence the formation of CC&H adaptation policy by determining (1) how the problem of CC&H is framed in the public and political discourse; (2) which political majorities for or against CC&H policies exist; and (3) what CC&H policy alternatives, barriers, and possibilities exist currently in Burkina Faso. This will provide an outlook and overview on the factors already in play in Burkina Faso’s policy process, and how best the process can be moved toward the goal of a formulated and implemented CC&H adaption policy.

## 5. Conclusions

Our study identified the knowledge of CC&H adaptation among SDPs in Burkina Faso by determining their perceptions of CC, of related health risks and vulnerabilities, and of CC impacts on agriculture and food security. SDPs are aware of the public health problems caused by CC and accurately perceive, in comparison to published literature, the health risks and vulnerabilities associated with extreme weather events and CC. The study has identified minor gaps in knowledge, which can be addressed with training for SDPs, and the study also illustrated the need for a surveilling system for groups of the population especially vulnerable to CC hazards. Overall, the study raises the question, *“where does Burkina Faso stand on the path of CC&H policy formulation and what are the current barriers in the process?”*, as our study has demonstrated that knowledge and awareness, common barriers in the beginning stages of policy formulation, are not relevant in Burkina Faso, and therefore are not currently barriers to formulating CC&H adaptation policy.

## Supplementary Materials

The following are available online at https://www.mdpi.com/article/10.3390/ijerph18094972/s1, Figure S1: Consolidated Criteria for Reporting Qualitative Research Checklist (COREQ).

## References

[1] McMichael A.J., Friel S., Nyong A., Corvalan C. (2008). Global environmental change and health: Impacts, inequalities, and the health sector. BMJ.

[2] McMichael A.J., Woodruff R.E., Hales S. (2006). Climate change and human health: Present and future risks. Lancet.

[3] UNFPA (2009). The State of World Population Report 2009: Facing a Changing World: Women, Population and Climate.

[4] UNDP (2007). Human Development Report 2007: Background Paper on Risks, Vulnerability and Adaptation in Bangladesh.

[5] Haines A., Kovatsa R.S., Campbell-Lendrumb D., Corvalanb C. (2006). Climate change and human health: Impacts, vulnerability and public health. J. R. Inst. Public Health.

[6] Stephenson J., Newman K., Mayhew S. (2010). Population dynamics and climate change: What are the links?. J. Public Health.

[7] Barnett J. (2003). Security and climate change. Glob. Environ. Chang..

[8] Tyagi S., Garg N., Paudel R. (2014). Environmental degradation: Causes and consequences. Eur. Res..

[9] Woodward A., Smith K. (2014). Chapter 11. Human Health: Impacts, Adaptation, and Co-Benefits. IPCC WGII AR5.

[10] IPCC (2018). Summary for Policymakers. Global Warming of 1.5 °C. An IPCC Special Report on the Impacts of Global Warming of 1.5 °C above Pre-Industrial Levels and Related Global Greenhouse Gas Emission Pathways, in the Context of Strengthening the Global Response to the Threat of Climate Change, Sustainable Development, and Efforts to Eradicate Poverty.

[11] IPCC (2012). Glossary of terms. Managing the Risks of Extreme Events and Disasters to Advance Climate Change Adaptation.

[12] Wittig R., König K., Schmidt M., Szarzynski J. (2007). A study of climate change and anthropogenic impacts in West Africa. Environ. Sci. Pollut. Res.-Int..

[13] Kotir J.H. (2011). Climate change and variability in Sub-Saharan Africa: A review of current and future trends and impacts on agriculture and food security. Environ. Dev. Sustain..

[14] UNFCCC United Nations Framework Convention On Climate Change Parties & Observers. https://unfccc.int/parties-observers.

[15] Sorgho R., Mank I., Kagoné M., Souares A., Danquah I., Sauerborn R. (2020). We will always ask ourselves the question of how to feed the family: Subsistence farmers perceptions on adaptation to climate change in Burkina Faso. Int. J. Environ. Res. Public Health.

[16] Laux P.W.S., Wagner A., Jacobeit J., Bardossy A., Kunstmann H. (2009). Modelling daily precipitation features in the Volta basin of West Africa. Int. J. Climatol..

[17] Barry A.A., Caesar J., Klein Tank A.M.G., Aguilar E., McSweeney C., Cyrille A.M., Nikiema M.P., Narcisse K.B., Sima F., Stafford G. (2018). West Africa climate extremes and climate change indices. Int. J. Climatol..

[18] Ibrahim B., Karambiri H., Polcher J., Yacouba H., Ribstein P. (2014). Changes in rainfall regime over Burkina Faso under the climate change conditions simulated by 5 regional climate models. Clim. Dyn..

[19] Hondula D.M., Rocklöv J., Sankoh O.A. (2012). Past, present and future climate at select INDEPTH member Health and Demographic Surveillance Systems in Africa and Asia. Glob. Health Action.

[20] Burkina Faso, Ministry of Environment (2016). DECRET N° 2016­383/PRES/PM/MEEVCC. N° 2016­383.

[21] Rowhani P., Lobell D.B., Linderman M., Ramankutty N. (2011). Climate variability and crop production in Tanzania. Agric. For. Meteorol..

[22] Haines A., Ebi K. (2019). The Imperative for Climate Action to Protect Health. N. Engl. J. Med..

[23] Watts N., Amann M., Arnell N., Ayeb-Karlsson S., Belesova K., Berry H., Bouley T., Boykoff M., Byass P., Cai W. (2018). The 2018 report of the Lancet Countdown on health and climate change: Shaping the health of nations for centuries to come. Lancet.

[24] Bunker A. Effects of ambient temperature on non-communicable disease health outcomes in vulnerable populations. Heidelberg University 2018. https://archiv.ub.uni-heidelberg.de/volltextserver/25388/.

[25] Bunker A., Wildenhain J., Vandenbergh A., Henschke N., Rocklöv J., Hajat S., Sauerborn R. (2016). Effects of air temperature on climate-sensitive mortality and morbidity outcomes in the elderly; a systematic review and meta-analysis of epidemiological evidence. EBioMedicine.

[26] Kjellstrom T., Briggs D., Freyberg C., Lemke B., Otto M., Hyatt O. (2016). Heat, Human Performance, and Occupational Health: A Key Issue for the Assessment of Global Climate Change Impacts. Annu. Rev. Public Health.

[27] Williams M., Feldscher K. (2016). Putting a Human Face on Climate Change.

[28] Belesova K., Gasparrini A., Sié A., Sauerborn R., Wilkinson P. (2017). Household cereal crop harvest and children’s nutritional status in rural Burkina Faso. Environ. Health.

[29] Belesova K., Gasparrini A., Sié A., Sauerborn R., Wilkinson P. (2017). Annual Crop-Yield Variation, Child Survival, and Nutrition Among Subsistence Farmers in Burkina Faso. Am. J. Epidemiol..

[30] Nelson G.C., Rosegrant M.W., Palazzo A., Gray I., Ingersoll C., Robertson R., Tokgoz S., Zhu T., Sulser T.B., Ringler Msangi S. (2010). Food Security, Farming, and Climate Change to 2050: Scenarios, Results, Policy Options.

[31] Niang I., Ruppel O.C., Abdrabo M.A., Essel A., Lennard C., Padgham J., Urquhart P. (2014). Africa. Climate Change 2014: Impacts, Adaptation, and Vulnerability. Part B: Regional Aspects, Contribution of Working Group II to the Fifth Assessment Report of the Intergovernmental Panel on Climate Change.

[32] De Longueville F., Hountondji Y.-C., Kindo I., Gemenne F., Ozer P. (2016). Long-term analysis of rainfall and temperature data in Burkina Faso (1950–2013). Int. J. Climatol..

[33] Karst I.G., Mank I., Traoré I., Sorgho R., Stückemann K.-J., Simboro S., Sié A., Franke J., Sauerborn R. (2020). Estimating yields of household fields in rural subsistence farming systems to study food security in burkina faso. Remote Sens..

[34] Sorgho R., Quiñonez C.A.M., Louis V.R., Winkler V., Dambach P., Sauerborn R., Horstick O. (2020). Climate Change Policies in 16 West African Countries: A Systematic Review of Adaptation with a Focus on Agriculture, Food Security, and Nutrition. Int. J. Environ. Res. Public Health.

[35] Kalame F.B., Kudejira D., Nkem J. (2011). Assessing the process and options for implementing National Adaptation Programmes of Action (NAPA): A case study from Burkina Faso. Mitig. Adapt. Strateg. Glob. Chang..

[36] Sherman M.H., Ford J. (2014). Stakeholder engagement in adaptation interventions: An evaluation of projects in developing nations. Clim. Policy.

[37] Chin-Yee S. (2018). Defining Climate Policy in Africa: Kenya Climate Change Policy Processes.

[38] Haas P.M., Stevens C. (2011). Organized science, usable knowledge, and multilateral environmental governance. Gov. Air Dyn. Sci. Policy Citiz. Interact..

[39] Koch I.C., Vogel C., Patel Z. (2007). Institutional dynamics and climate change adaptation in South Africa. Mitig. Adapt. Strateg. Glob. Chang..

[40] Roberts D. (2008). Thinking globally, acting locally—Institutionalizing climate change at the local government level in Durban, South Africa. Environ. Urban..

[41] Kondracki N.L., Wellman N.S., Amundson D.R. (2002). Content analysis: Review of methods and their applications in nutrition education. J. Nutr. Educ. Behav..

[42] Hsieh H.F., Shannon S.E. (2005). Three approaches to qualitative content analysis. Qual. Health Res..

[43] McMahon S.A., Winch P.J. (2018). Systematic debriefing after qualitative encounters: An essential analysis step in applied qualitative research. BMJ Glob. Health.

[44] Saldaña J. (2009). The Coding Manual for Qualitative Researchers.

[45] Miles M.B., Huberman A.M. (1994). Qualitative Data Analysis.

[46] Polkinghorne D.E. (2007). Validity Issues in Narrative Research. Qual. Inq..

[47] Booth A., Hannes K., Harden A., Noyes J., Harris J., Tong A. (2014). COREQ (consolidated criteria for reporting qualitative studies). Guidelines for Reporting Health Research: A User’s Manual.

[48] Tong A., Sainsbury P., Craig J. (2007). Consolidated criteria for reporting qualitative research (COREQ): A 32-item checklist for interviews and focus groups. Int. J. Qual. Health Care.

[49] Carter N., Bryant-Lukosius D., DiCenso A., Blythe J., Neville A.J. (2014). The use of triangulation in qualitative research. Oncol. Nurs. Forum.

[50] Thurmond V.A. (2001). The point of triangulation. J. Nurs. Scholarsh..

[51] Shenton A.K. (2004). Strategies for ensuring trustworthiness in qualitative research projects. Educ. Inf..

[52] Anderson C. (2010). Presenting and Evaluating Qualitative Research. Am. J. Pharm. Educ..

[53] Sandelowski M. (1994). Focus on qualitative methods. The use of quotes in qualitative research. Res. Nurs. Health.

[54] USGCRP (2016). The Impacts of Climate Change on Human Health in the United States: A Scientific Assessment. Crimmins.

[55] FAO (2001). The State of Food Insecurity in the World 2001.

[56] FAO (2018). The Future of Food and Agriculture: Alternative Pathways to 2050.

[57] FAO (2018). Combining Agricultural Biodiversity, Resilient Ecosystems, Traditional Farming Practices and Cultural Identity.

[58] INSD (2019). Annuaire Statistique 2018.

[59] Compaoré N. (2005). Burkina Faso: Recruiting women for legislative elections. Women in Parliament: Beyond Numbers.

[60] Mitsubishi UFJ Research and Consulting (2013). Country Gender Profile: Burkina Faso.

[61] Florida U.O. (2016). Gender Quotas and Representations: Burkina Faso.

[62] Dwyer S.C., Buckle J.L. (2009). The Space Between: On Being an Insider-Outsider in Qualitative Research. Int. J. Qual. Methods.

[63] van Nes F., Abma T., Jonsson H., Deeg D. (2010). Language differences in qualitative research: Is meaning lost in translation?. Eur. J. Ageing.

[64] Osaka S., Bellamy R. (2020). Natural variability or climate change? Stakeholder and citizen perceptions of extreme event attribution. Glob. Environ. Chang..

[65] Hosking J., Campbell-Lendrum D. (2012). How well does climate change and human health research match the demands of policymakers? A scoping review. Environ. Health Perspect..

[66] Fischer H., van den Broek K.L., Ramisch K., Okan Y. (2020). When IPCC graphs can foster or bias understanding: Evidence among decision-makers from governmental and non-governmental institutions. Environ. Res. Lett..

[67] Alam G.M.M., Alam K., Mushtaq S. (2017). Climate change perceptions and local adaptation strategies of hazard-prone rural households in Bangladesh. Clim. Risk Manag..

[68] Roy M., Cawood S., Hordijk M., Hulme D. (2016). Urban Poverty and Climate Change: Life in the Slums of Asia, Africa and Latin America.

[69] Admassie A., Adenew B., Tadege A. (2008). Perceptions of Stakeholders on Climate Change and Adaptation Strategies in Ethiopia.

[70] Tetteh E., Opareh N., Ampadu R., Antwi K.B. (2014). Impact of climate change: Views and perceptions of policy makers on smallholder agriculture in Ghana. Int. J. Sci. Basic Appl. Res..

[71] Walt G. (1994). Health Policy: An Introduction to Process and Power.

[72] Béland D., Howlett M. (2016). The Role and Impact of the Multiple-Streams Approach in Comparative Policy Analysis. J. Comp. Policy Anal. Res. Pract..

[73] Board E., WHO (2008). Resolution on Climate Change and Health. Provisional Agenda Item 4.

[74] WHO (2003). Climate Change and Human Health: Risk and Responses.

[75] Zizka A., Thiombiano A., Dressler S., Nacoulma B.M.I., Ouédraogo A., Ouédraogo I., Ouédraogo O., Zizka G., Hahn K., Schmidt M. (2015). Traditional plant use in Burkina Faso (West Africa): A national-scale analysis with focus on traditional medicine. J. Ethnobiol. Ethnomed..

[76] WHO (2015). Connecting Global Priorities: Biodiversity and Human Health.

[77] UCSUSA (2018). The Science Connecting Extreme Weather to Climate Change.

[78] Asare-Kyei D., Renaud F.G., Kloos J., Walz Y., Rhyner J. (2017). Development and validation of risk profiles of West African rural communities facing multiple natural hazards. PLoS ONE.

[79] Challinor A.J., Adger W.N., Benton T.G., Conway D., Joshi M., Frame D. (2018). Transmission of climate risks across sectors and borders. Philos. Trans. R. Soc. A Math. Phys. Eng. Sci..

[80] Berry H., Bowen K., Kjellstrom T. (2010). Climate change and mental health: A causal pathways framework. Int. J. Public Health.

[81] Cianconi P., Betrò S., Janiri L. (2020). The impact of climate change on mental health: A systematic descriptive review. Front. Psychiatry.

[82] Hayes K., Blashki G., Wiseman J., Burke S., Reifels L. (2018). Climate change and mental health: Risks, impacts and priority actions. Int. J. Ment. Health Syst..

[83] Acharibasam J.W., Anuga S.W. (2018). Psychological distance of climate change and mental health risks assessment of smallholder farmers in Northern Ghana: Is habituation a threat to climate change?. Clim. Risk Manag..

[84] FAO, IFAD, UNICEF, WFP, WHO (2018). The State of Food Security and Nutrition in the World 2018: Building Climate Resilience for Food Security and Nutrition.

[85] FAO (2013). Food Security and Agricultural Mitigation in Developing Countries: Options for Capturing Synergies.

[86] Ayodotun B., Bamba S., Adio A. (2019). Vulnerability Assessment of West African Countries to Climate Change and Variability. J. Geosci. Environ. Prot..

[87] GH2 (2010). Integrating Climate Change and Disaster Risk Reduction into National Developement Policies and Planning in Ghana.

[88] Beg N., Morlot J.C., Davidson O., Afrane-Okesse Y., Tyani L., Denton F., Sokona Y., Thomas J.P., La Rovere E.L., Parikh J.K. (2002). Linkages between climate change and sustainable development. Clim. Policy.

[89] Denton F. (2010). Financing adaptation in Least Developed Countries in West Africa: Is finance the real deal?. Clim. Policy.

